# Exercise triggers integrated lipid, sphingolipid and purine metabolic alterations in midlife women

**DOI:** 10.3389/fphys.2026.1871948

**Published:** 2026-08-05

**Authors:** Shamma Almuraikhy, Maha Sellami, Khaled Naja, Najeha Anwardeen, Haya Al-Sulaiti, Husam Eldin Elhag Abugabr Elhag, Mohamed A. Elrayess

**Affiliations:** 1 Biomedical Research Center, QU Health, Qatar University, Doha, Qatar; 2 Sport Coaching Department, College of Sport Sciences, Qatar University, Doha, Qatar; 3 Department of Biomedical Sciences, College of Health Sciences, QU Health, Qatar University, Doha, Qatar; 4 College of Medicine, QU Health, Qatar University, Doha, Qatar

**Keywords:** cardiometabolic health, exercise metabolomics, midlife women, moderate-intensity aerobic training, physical activity biomarkers

## Abstract

**Background:**

Regular moderate physical activity helps prevent cardiometabolic diseases, yet the underlying molecular pathways, particularly in women in early to mid-adulthood, remain incompletely understood. Women aged 30–50 years undergo hormonal and metabolic transitions that may modify both cardiometabolic risk and responsiveness to exercise. This study investigated exercise-induced changes in serum metabolic signatures in women aged 30–50 years following 8 weeks of moderate-intensity aerobic training (MAT).

**Method:**

Thirty-nine healthy pre-menopause females (30–50 years) with a BMI of 26.5–33.5 kg/m^2^ completed an 8-week supervised moderate-intensity aerobic exercise program. Fasting blood samples were collected before and after the intervention. Serum targeted quantitative metabolic profiling and clinical traits were assessed.

**Results:**

Univariate analysis revealed a coordinated post-exercise upregulation of dihydroceramides, glycosylceramides, ceramides, acylcarnitines (e.g., C14:2, C3-DC/C4-OH), diacylglycerols(DG 16:0_20:4, DG O-18:2_18:2),triacylglycerols, selected cholesterol esters (e.g., CE 20:3), cystine, and the nucleobase-related metabolite xanthine, alongside decreases in specific phosphatidylcholines species (PC O-30:2, PC O-28:0, PC O-30:0, PC 42:2, LPC 26:0), the amino acid serine, and CE 14:0,decrease in DG 18:1_18:1, CE 14:1, CE 14:0 and the amino acid–related metabolite cystine. Post-exercise correlation analysis identified distinct associations between metabolic profiles and clinical traits, with higher physical activity (metabolic equivalent of task, MET) positively linked to selected ceramide species (Cer d18:1/16:0, Cer d18:2/18:0).

**Conclusion:**

Moderate aerobic exercise in midlife women elicits a coordinated serum metabolomic response characterized by remodeling of lipid and sphingolipid pathways, increased fatty acid related mitochondrial handling, and elevated energy/redox turnover. The upregulation of dihydroceramides, glycosylceramides, ceramides, acylcarnitines, glycerolipids, cholesterol esters, and xanthine, together with reductions in specific phosphatidylcholines, cystine and serine, supports an exercise-induced “lipid sphingolipid energy stress” signature. Distinct associations of ceramide, glyceride, and purine species with MET, insulin indices, BMI, HDL, and catalase further suggest that these metabolites are promising candidate markers of exercise adaptation in midlife women.

## 1 Introduction

Regular moderate physical activity is recognized as one of the most effective non-pharmacological strategies for promoting cardiometabolic health and longevity. It substantially reduces the risk of obesity, type 2 diabetes, and cardiovascular disease through systemic improvements in glucose and lipid metabolism, vascular function, and chronic inflammation control (Guo and Wang, 2025; Kistler et al., 2026). Exercise induces coordinated physiological adaptations across multiple organ systems, including skeletal muscle, adipose tissue, the liver, and the cardiovascular system, thereby improving energy homeostasis and overall metabolic efficiency. These benefits are well documented in epidemiological and interventional studies that consistently demonstrate dose-dependent relationships between physical activity and cardiometabolic protection (Zhao et al., 2025; Kistler et al., 2026).

Despite extensive research, the molecular mechanisms underlying the beneficial effects of moderate-intensity aerobic exercise remain incompletely defined. This gap is particularly evident among women in early to mid-adulthood (30–50 years), a stage characterized by pronounced endocrine and metabolic fluctuations preceding menopause. Even in healthy, non-obese individuals, these hormonal transitions correspond with shifts in lipid and amino acid metabolism, manifesting as more atherogenic lipoprotein profiles, elevated triglycerides, reduced HDL-C, and increased branched-chain amino acids traits linked to early atherogenesis and insulin resistance (Wu et al., 2023).

Moderate aerobic exercise during this life stage has been shown to mitigate these adverse trajectories by improving classical biomarkers such as triglycerides, total cholesterol, HDL-C, and insulin sensitivity (Bernal et al., 2025). Beyond conventional clinical markers, however, exercise elicits widespread changes in cellular energy metabolism, mitochondrial turnover, oxidative stress regulation, and substrate utilization. These transformations are driven by molecular signaling networks involving AMP-activated protein kinase (AMPK), peroxisome proliferator-activated receptors (PPARs), and cytokine cross-talk between muscle and adipose tissue (Tero-Vescan et al., 2025). Yet, despite these mechanistic insights, gaps remain in understanding how moderate-intensity aerobic training typical of recommended public health guidelines remodels systemic metabolism in women and what specific metabolite signatures best reflect these adaptive processes.

Metabolomics has emerged as a powerful platform for elucidating the biochemical pathways influenced by exercise. By simultaneously quantifying hundreds of metabolites, metabolomics captures dynamic alterations in amino acid metabolism, lipid remodeling, and mitochondrial substrate flux that conventional biochemical assays cannot detect (Jaguri et al., 2023). Among available approaches, targeted quantitative metabolomics provides the additional advantage of absolute concentration quantification, enabling inter-study comparability and correlation with physiological parameters (McMillen et al., 2023).

Despite these advances, a predominance of male-dominants persist, restricting our understanding of sex-specific exercise adaptations at the molecular level (Lew et al., 2022). Disentangling these complexities requires sex-balanced study designs and age-stratified analyses that reflect the biological variability inherent to women’s physiology. Clarifying such patterns holds major implications for personalized exercise prescriptions, early biomarker discovery, and preventive strategies against cardiometabolic disease in women. Therefore, the present study investigates integrated metabolic adaptations to an eight-week moderate-intensity aerobic exercise intervention in healthy women aged 30–50 years.

## 2 Method

### 2.1 Study participants

Thirty-nine apparently healthy, pre-menopause women aged 30–50 years were recruited for this study. Inclusion criteria included the absence of cardiovascular disease, type 2 diabetes, musculoskeletal disorders, thrombotic conditions, or neurological disorders. All participants provided written informed consent prior to participation. The study protocols were approved by Qatar University (QU-IRB 1798-EA/23) in compliance with the regulations of the Qatar Ministry of Public Health.

### 2.2 Training session

Participants undertook an 8-week aerobic exercise intervention aligned with established recommendations from American College of Sports Medicine (ACSM) and American Heart association (AHA) (Samjoo et al., 2013; Marson et al., 2016; Nomura et al., 2019; Battista et al., 2021; Kanaley et al., 2022). Exercise intensity was progressively increased over the training period, beginning at 40–60% of maximal heart rate (HRmax) and approximately 50% of VO_2_ peak, and reaching 60–70% intensity by the final week. Sessions were performed three times per week, each lasting 50 minutes. Daily physical activity levels were assessed using the International Physical Activity Questionnaire (IPAQ), from which Metabolic Equivalent of Task (MET) values were derived. These values were applied to standardize exercise intensity and estimate energy expenditure across participants with differing body masses.

### 2.3 Study measures

Body composition was assessed before and after the training period using a TANITA analyzer (Tanita Corporation, Japan). Measurements were taken in the early morning. The device provided data on body weight (kg) and body mass index (BMI) (Heymsfield et al., 2024).

### 2.4 Clinical parameters and anti-oxidant enzymes activity measurements

Fasting blood samples were obtained before the 8-week intervention period and forty-eight hours following last training session. Fasting glucose, total cholesterol, triglycerides, HDL, and LDL concentrations were analyzed using a Mindray BS240 clinical chemistry analyzer in accordance with the manufacturer’s protocols. Serum insulin levels were measured using a commercial ELISA kit (Mercodia, UK) following the manufacturer’s instructions. Insulin resistance was estimated using the Homeostatic Model Assessment of Insulin Resistance (HOMA-IR), calculated as: fasting glucose (mmol/L) × fasting insulin (mIU/mL)/22.5. The activities of antioxidant enzymes, including superoxide dismutase and catalase, were determined using colorimetric assay kits (EIASODC and EIACATC, respectively; Thermo Fisher Scientific, USA) according to the manufacturer’s guidelines.

### 2.5 Metabolomics analysis

Targeted metabolomics analysis of serum samples was performed using the MxP^®^ Quant 500 kit (Biocrates, Innsbruck, Austria), which evaluates 630 metabolites across 26 biochemical classes. A volume of 10 μL from the blank solution (phosphate-buffered saline), calibration standard solutions, quality control solutions, and each human serum sample was separately added to a 96-well plate. Each well contained a filter disk pre-loaded with internal standards provided by Biocrates. The plate was then dried for 30 minutes under nitrogen flow using a TurboVap^®^96 Dual (Biotage). After drying, a derivatization solution containing 5% phenyl isothiocyanate was added to all wells, and the plate was incubated for 60 minutes at room temperature, followed by another drying step with the TurboVap^®^96 Dual. Subsequently, 300 μL of extract solvent (5 mM ammonium acetate in methanol) was added to each well, and the plate was shaken at room temperature for 30 minutes, then centrifuged for 2 minutes to elute the extract into the lower capture plate. Extracts were divided into LC and FIA dilution plates following the Biocrates protocol. The extracts were analyzed using FIA-MS/MS and LC-MS/MS methods on a platform consisting of a Waters Xevo TQS triple quadrupole mass spectrometer integrated with a Waters Acquity UHPLC (Waters, Milford, MA) with the employment of multiple reaction monitoring (MRM) to detect the analytes. The LC column was an MxP^®^ Quant 500 kit system column (included with the Biocrates kit). Mobile phase compositions, temperature, and LC gradient elution conditions were set according to the Biocrates methods. Concentrations were determined using appropriate mass spectrometry software, and the data were imported into Biocrates MetIDQ software for further analysis. Data processing, validation, quantification, and export were performed using WebIDQ cloud software (Biocrates, Innsbruck, Austria), and the concentrations (μM) for each metabolite within different classes were calculated.

### 2.6 Statistical analysis

Metabolomics data were inspected for missingness at both the subject and feature level. For each sample, the proportion of missing metabolite values was computed; all subjects had <90% missingness and therefore all were retained for downstream analysis. Metabolite-level missingness was then evaluated, and features with ≥60% missing values were removed prior to modelling. Remaining missing values were imputed using a feature-wise half-minimum method, where missing entries were replaced with one-half of the lowest observed value for that metabolite. Metabolites exhibiting zero variance across samples were also removed. The pre-processed dataset was used to construct the predictor matrix X, the outcome vector Y (Activity), and the subject identifier vector ID, which served as the multilevel factor for multilevel PCA and multilevel PLS-DA analyses.

To characterize overall metabolic variation while accounting for repeated measures, a multilevel principal component analysis (ml-PCA) was performed using R *mixOmics*, with the subject ID specified in the multilevel design matrix. Scores on PC1 and PC2 were extracted to visualize sample structure and between-group separation. Multilevel partial least-squares discriminant analysis (ml PLS-DA) was performed to identify metabolic patterns associated with the intervention while accounting for the paired study design. Participant ID was specified as the multilevel factor, enabling the analysis to focus on within-participant variation between the pre- and post-intervention measurements. A two-component multilevel PLS-DA was fitted. Model performance and robustness were assessed using 7-fold cross-validation with 200 repetitions. Classification error was assessed using the maximum, centroid and Mahalanobis distance prediction methods. Model performance and number of components were evaluated using the balanced error rates, and balanced accuracy was calculated as one minus balanced error rate. Model performance assessment indicated low error rates for both components (0.083 for component 1 and 0.071 for component 2), with small standard deviations across resampling iterations (0.021 for component 1 and 0.019 for component 2), supporting model stability and low risk of overfitting. Variable importance in projection (VIP) scores was used to summarize the contribution of individual metabolites to group discrimination.

Univariate associations between each metabolite and activity were tested using linear mixed-effects models (LMMs) fitted separately for each metabolite using the R *emmeans* package. Models were of the form metabolite ~ activity + covariates + (1 | ID), adjusting for BMI, HOMA-IR, and principal component 1. PC1 was included to account for broad systemic variation across the metabolomic measurements, including potential technical and residual sample level variation. Sensitivity analyses were performed by repeating all metabolite-specific mixed effects models without adjustment for PC1. For each metabolite, the fixed-effect estimate, standard error, and p-value for the activity coefficient were extracted. Multiple testing correction was applied using the Benjamini–Hochberg false discovery rate (FDR) procedure where appropriate. All analysis and visualizations were implemented in R (v.4.2.1).

## 3 Results

### 3.1 General characteristics of participants of study population

**Table 1** presents the general characteristics of the study population. All participants were aged 30–50 years and the cohort included both overweight and obese participants with a median BMI of 30.35 kg/m^2^ and an interquartile range of 26.525–33.525 kg/m^2^. Participants showed moderate but significant BMI decrease after the intervention. As expected, MET increased significantly with exercise (p value=0.005). Fasting blood glucose, insulin, and catalase levels did not change following physical training. In contrast, SOD activity increased significantly after the intervention (p value=0.001). Lipid profile including total cholesterol, HDL, and LDL decreased significantly after exercise (p value=0.001, <0.001, and 0.003 respectively).

**Table 1:** General characteristics of participants.

Clinical Traits	Before (n=39)	After (n=39)	p-value
Age (yrs)	42.74 (7.28)		–
BMI(Body Mass Index)	30.35 (26.525-33.525)	30.05 (25.875-32.525)	0.041
Fasting Blood Sugar (mmol/L)	5.3 (5.2-5.85)	5.3 (5-5.95)	0.220
Insulin (mU/L)	9.37 (5.955-16.33)	9.01 (5.44-21.865)	0.878
HOMA-IR	2.27 (1.365-4.34)	2.26 (1.355-6.055)	0.572
Catalase (u/ml)	20.43 (17.56-25.64)	22.955 (18.0025-26.72)	0.210
SOD.u.ml	0.699 (0.636-0.72875)	0.7695 (0.694-0.911)	0.001
Total Cholesterol (mmol/L)	4.64 (3.94-5.16)	4.01 (3.445-4.575)	0.001
Triglyceride (mmol/L)	0.88 (0.655-1.295)	0.81 (0.625-1.365)	0.620
High density lipoprotein HDL (mmol/L)	1.36 (0.36)	1.17 (0.32)	0.000
Low density lipoprotein LDL (mmol/L)	2.85 (2.59-3.515)	2.66 (2.205-3.19)	0.003
Walk (min/week)	594 (255.75-1237.5)	594 (231-1151)	0.6335
Vigorous (min/week)	0 (0-1260)	0 (0-120)	0.5135
Moderate (min/week)	320 (0-720)	600 (280-1320)	0.021
Total MET value/week	1386 (498-2475)	2160 (1027-3765)	0.005
Weight (Kg)	74.8 (67.05-86.3)	73.75 (66.475-79.55)	0.240

Data are presented as mean (SD)/median (IQR) after assessing for normality using Shapiro Wilk’s test. The values of before and after were compared using paired Student’s t test/Wilcoxon sum of ranks. A p-value of <0.05 was considered significant. MET (Metabolic Equivalent of Task).

### 3.2 Exercise-induced metabolic signatures: multivariate modeling

**Figure 1:**
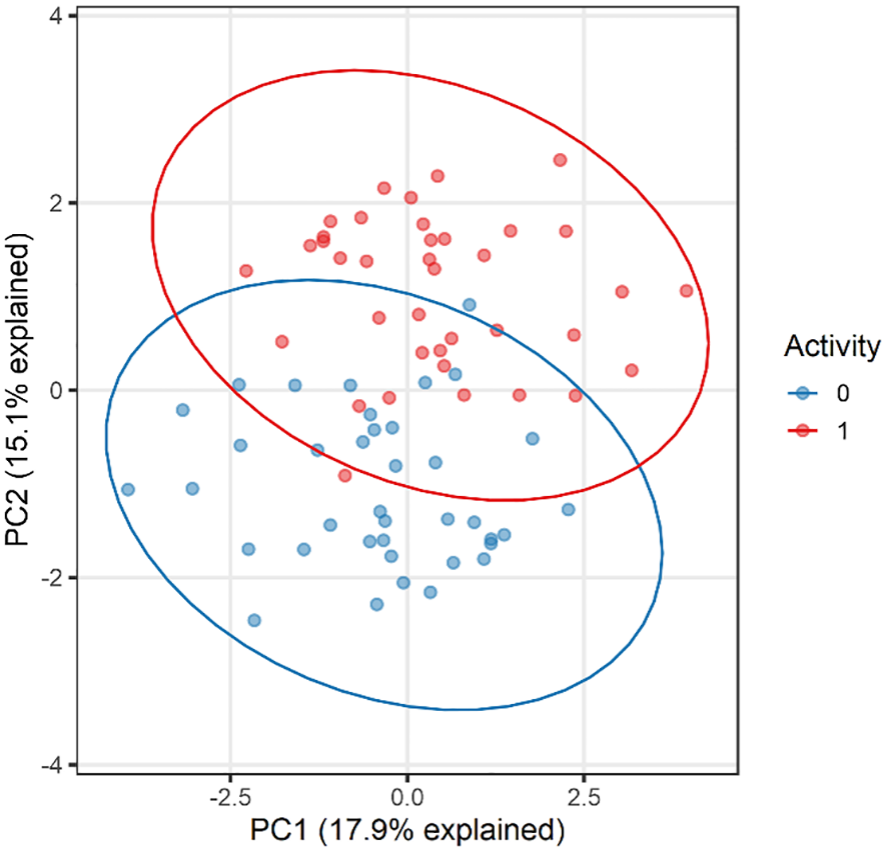
Scores from the multilevel principal component analysis (ml-PCA) are shown for PC1 (17.9% variance explained) and PC2 (15.1% variance explained), with Activity groups (0 = before, 1 = after) indicated by colour. Each point represents an individual sample, and ellipses denote the 95% confidence regions for each group.

Targeted metabolomics was applied to compare the metabolic signatures of 39 participants before and after exercise intervention. PCA was performed based on all retained metabolites after pre-processing to capture the global view of the data. PC1 showed no clear separation between the two groups, PC2 explained 15% of the total variance in the data (**Figure 1**). We removed PC2 from the univariate analysis due to its role as a possible confounder. No other batch effects were seen in our data.

### 3.3 Multilevel partial least squares-discriminant analyses

**Figure 2:**
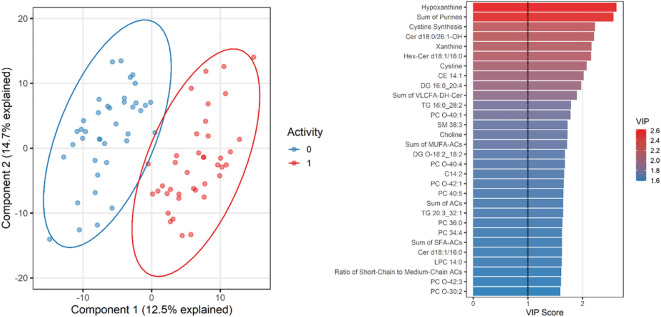
(left) Scores from the multilevel pls-da are shown for components 1 and 2, with subject ID included as the multilevel factor to remove within-subject variation. Each point represents a sample, coloured by Activity group. Ellipses indicate the 95% confidence region for each group. (right) VIP scores from the mPLS-DA model are presented for the top 30 metabolites contributing to group discrimination. Higher VIP values indicate greater importance of a metabolite in the projection of samples onto the discriminant components. Bars are coloured by VIP magnitude.

Multilevel PLS-DA (ml-PLS-DA) (**Figure 2** left) was conducted to identify metabolite patterns that discriminate between the pre- and post-exercise states while accounting for the repeated-measures design. The first two components captured the major sources of between-subject variation related to activity. Model performance was evaluated using repeated 7-fold cross-validation with 200 resampling iterations, yielding balanced error rates, of 5.9% and a corresponding balanced accuracy of 94.1%. The balanced error rates obtained using the maximum and Mahalanobis distance methods were both 7.09%, corresponding to balanced accuracies of 91.9%. VIP scores (**Figure 2** right) were used to identify the metabolites contributing most strongly to group separation, and these features were subsequently examined in the univariate mixed-effects analysis.

### 3.4 Metabolite-specific responses to exercise training

To evaluate metabolite-specific differences before and after exercise, linear mixed-effects models were fitted for each metabolite with activity as the main predictor while accounting for repeated measurements using a random intercept for subject ID. Models were adjusted for BMI, HOMA-IR, and principal component 1. Several metabolites showed significant associations with activity after Benjamini–Hochberg FDR correction, indicating measurable metabolic shifts induced by the intervention. These metabolites (**Table 2**) largely overlapped with the top discriminative features identified in the ml-PLS-DA analysis, supporting the consistency of findings across multivariate and univariate approaches. Univariate analysis showed upregulation of several sub-pathways, including dihydroceramides (Cer d18:0/26:1-OH), acylcarnitines (C14:2, C3-DC/C4-OH), glycosylceramides (Hex-Cer d18:1/18:1, Hex-Cer d18:1/26:1, Hex3Cer d18:1/16:0, Hex-Cer d18:2/18:0), ceramides (Cer d18:1/16:0, Cer d18:2/18:0), diacylglycerols (DG 16:0_20:4, DG O-18:2_18:2, DG 18:1_18:1), triacylglycerols (TG 22:1_32:5, TG 16:0_38:6, TG 16:0_28:2), metabolic indicators, cholesterol esters (CE 14:1, CE 20:3, CE 14:0), and the nucleobase-related metabolite xanthine, together with lower levels of phosphatidylcholines (PC O-30:2, PC O-28:0, PC O-30:0, PC 42:2,PC 32:3, LPC 26:0, LPC 24:0). In addition to reduction in DG 18:1_18:1, CE 14:1, CE 14:0, cystine and the amino acid serine. Sensitivity analyses were subsequently conducted without adjustment for PC1 to evaluate whether its adjustment had attenuated intervention-related biological variation. The effect estimates and directions were broadly consistent between the PC1-adjusted and unadjusted models, and the principal metabolomic findings remained unchanged. Results are provided in **Supplementary Table 1**.

**Table 2:** Metabolites/metabolic indicators associated with activity assessed using linear mixed model.

Metabolite	Sub-pathway	Estimate	SE	p-value	FDR
Cer d18:0/26:1-OH	Dihydroceramides	0.612	0.072	3.92 × 10^−12^	1.94 × 10^−9^
CE 14:1	Cholesterol Esters	-0.028	0.003	1.08 × 10^−11^	2.67 × 10^−9^
Cystine Synthesis	Metabolic Indicators	-0.093	0.010	1.08 × 10^−10^	1.79 × 10^−8^
Hypoxanthine	Metabolic Indicators	0.430	0.057	1.30 × 10^−8^	1.41 × 10^−6^
Sum of MUFA-ACs	Metabolic Indicators	0.194	0.030	1.49 × 10^−8^	1.41 × 10^−6^
Sum of Purines	Metabolic Indicators	0.429	0.058	1.71 × 10^−8^	1.41 × 10^−6^
Cystine	Aminoacids Related	-0.457	0.064	2.24 × 10^−8^	1.58 × 10^−6^
DG 16:0_20:4	Diacylglycerols	0.055	0.008	7.05 × 10^−8^	4.36 × 10^−6^
Sum of ACs	Metabolic Indicators	0.841	0.141	1.06 × 10^−7^	5.84 × 10^−6^
DG O-18:2_18:2	Diacylglycerols	0.058	0.010	1.46 × 10^−7^	7.24 × 10^−6^
Sum of SFA-ACs	Metabolic Indicators	0.804	0.138	1.88 × 10^−7^	8.46 × 10^−6^
Carnitine Esterification	Metabolic Indicators	0.125	0.023	1.16 × 10^−6^	4.79 × 10^−5^
Sum of Medium-Chain ACs	Metabolic Indicators	0.285	0.055	2.30 × 10^−6^	8.77 × 10^−5^
C14:2	Acylcarnitines	0.098	0.019	2.58 × 10^−6^	9.11 × 10^−5^
Ratio of Short-Chain to Medium-Chain ACs	Metabolic Indicators	1.022	0.187	3.74 × 10^−6^	1.24 × 10^−4^
Xanthine	Nucleobases Related	0.262	0.049	7.12 × 10^−6^	2.20 × 10^−4^
Hex-Cer d18:1/18:1	Glycosylceramides	0.033	0.007	1.23 × 10^−5^	3.57 × 10^−4^
Cer d18:1/16:0	Ceramides	0.043	0.008	1.49 × 10^−5^	4.08 × 10^−4^
C3-DC (C4-OH)	Acylcarnitines	0.186	0.038	2.31 × 10^−5^	6.02 × 10^−4^
Hex-Cer d18:1/26:1	Glycosylceramides	0.027	0.006	3.32 × 10^−5^	8.22 × 10^−4^
Ratio of Medium-Chain to Long-Chain ACs	Metabolic Indicators	0.570	0.132	5.24 × 10^−5^	1.23 × 10^−3^
PC O-30:2	Phosphatidylcholines	-0.023	0.005	6.61 × 10^−5^	1.49 × 10^−3^
PC O-28:0	Phosphatidylcholines	-0.143	0.034	1.46 × 10^−4^	3.15 × 10^−3^
LPC 26:0	Phosphatidylcholines	-0.159	0.039	2.36 × 10^−4^	4.87 × 10^−3^
PC O-30:0	Phosphatidylcholines	-0.023	0.006	2.67 × 10^−4^	5.28 × 10^−3^
PC 42:2	Phosphatidylcholines	-0.004	0.001	3.58 × 10^−4^	6.82 × 10^−3^
Anserine Synthesis	Metabolic Indicators	0.099	0.027	5.22 × 10^−4^	9.57 × 10^−3^
Sum of VLCFA-DH-Cer	Metabolic Indicators	0.357	0.093	5.71 × 10^−4^	1.01 × 10^−2^
Asn Synthesis	Metabolic Indicators	-0.159	0.042	6.47 × 10^−4^	1.08 × 10^−2^
Sum of MUFA-DGs	Metabolic Indicators	0.181	0.048	6.55 × 10^−4^	1.08 × 10^−2^
Sum of VLCFA-LPCs	Metabolic Indicators	-0.159	0.044	8.04 × 10^−4^	1.28 × 10^−2^
TG 22:1_32:5	Triacylglycerols	0.002	0.001	9.60 × 10^−4^	1.49 × 10^−2^
Hex3Cer d18:1/16:0	Glycosylceramides	-0.041	0.011	1.01 × 10^−3^	1.52 × 10^−2^
LPC 24:0	Phosphatidylcholines	-0.067	0.019	1.12 × 10^−3^	1.64 × 10^−2^
Taurine Conjugation of DCA	Metabolic Indicators	0.050	0.015	1.40 × 10^−3^	1.98 × 10^−2^
PC 32:3	Phosphatidylcholines	-0.022	0.006	1.55 × 10^−3^	2.13 × 10^−2^
DG 18:1_18:1	Diacylglycerols	-0.084	0.026	2.42 × 10^−3^	3.17 × 10^−2^
Ser	Aminoacids	-0.087	0.026	2.44 × 10^−3^	3.17 × 10^−2^
Hex-Cer d18:1/16:0	Glycosylceramides	0.086	0.026	2.61 × 10^−3^	3.30 × 10^−2^
CE 20:3	Cholesterol Esters	0.201	0.062	2.67 × 10^−3^	3.30 × 10^−2^
Sum of VLCFA-Cer	Metabolic Indicators	-0.427	0.139	3.07 × 10^−3^	3.70 × 10^−2^
TG 16:0_38:6	Triacylglycerols	0.031	0.009	3.14 × 10^−3^	3.70 × 10^−2^
Cer d18:2/18:0	Ceramides	0.013	0.004	3.35 × 10^−3^	3.85 × 10^−2^
b-Ala Synthesis	Metabolic Indicators	-0.280	0.090	3.51 × 10^−3^	3.87 × 10^−2^
Sum of SFA-DGs	Metabolic Indicators	0.014	0.005	3.60 × 10^−3^	3.87 × 10^−2^
Hex-Cer d18:2/18:0	Glycosylceramides	0.023	0.008	3.77 × 10^−3^	3.92 × 10^−2^
CE 14:0	Cholesterol Esters	-0.208	0.067	3.80 × 10^−3^	3.92 × 10^−2^
TG 16:0_28:2	Triacylglycerols	0.103	0.035	4.60 × 10^−3^	4.65 × 10^−2^

### 3.5 Metabolic pathways associated with physical activity

**Figure 3:**
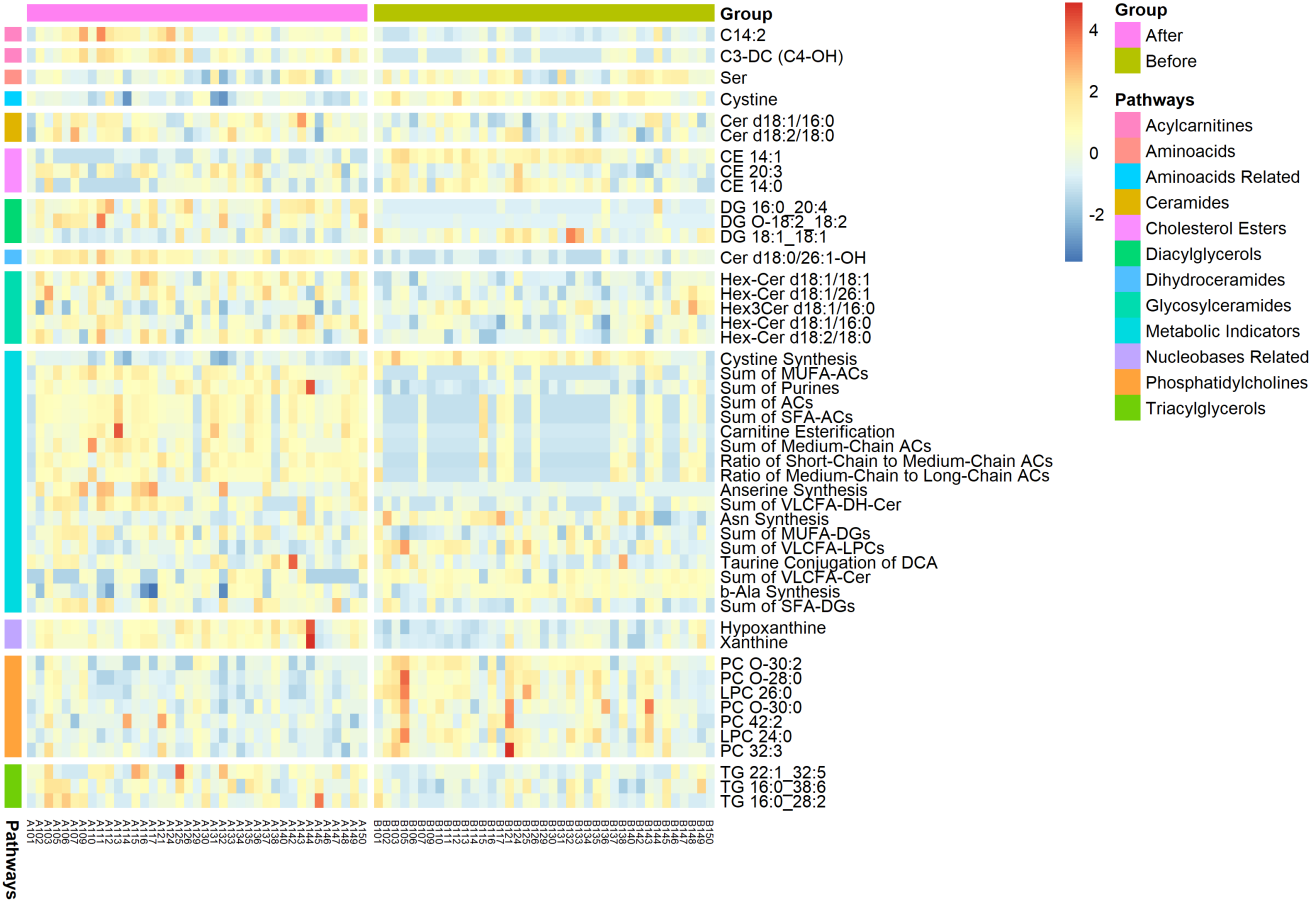
Heatmap showing the FDR significant metabolic pathways associated with activity after intervention using linear mixed model for each metabolite, adjusting for BMI, HOMA-IR, and principal component 1.

The heat-map (**Figure 3**) displays FDR-significant metabolic pathways associated with physical activity after the intervention, based on linear mixed-model analyses for each metabolite, adjusting for BMI, HOMA-IR, and principal component 1.

### 3.6 Correlation of metabolites with clinical traits

**Figure 4:**
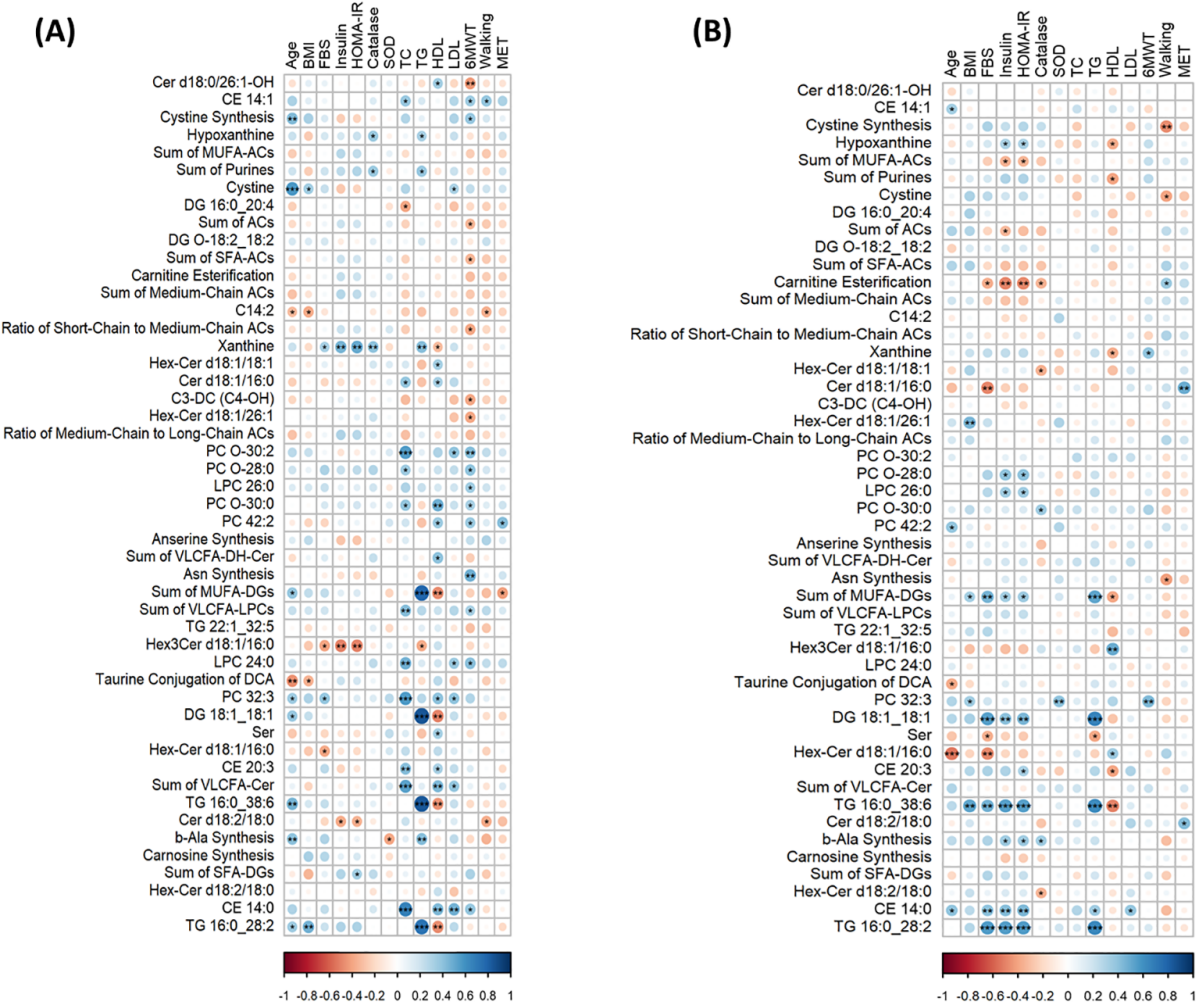
Correlation matrix showing the positive/negative (blue/red) correlation between the metabolites and clinical traits before intervention **(A)** and after exercise **(B)**. Significant correlations are depicted by ***/**/* denoting <0.001/<0.01/<0.05.

Post-exercise correlation analysis revealed distinct metabolic signatures associated with clinical traits (**Figures 4A, B**). MET positively correlated with ceramide species Cer d18:1/16:0 (and Cer d18:2/18:0. Insulin, FBS, and HOMA-IR showed positive correlations with hypoxanthine, PC O-28:0, LPC 26:0, DG 18:1_18:1, TG 16:0_38:6, CE 14:0, and TG 16:0_28:2, but negatively correlated with carnitine esterification. BMI positively correlated with hexosyl-ceramide d18:1/26:1, sum of MUFA-DGs, PC 32:3, and TG 16:0_38:6. HDL positively correlated with hexosyl-ceramides d18:1/16:0 species but negatively with hypoxanthine, purines, xanthine, MUFA-DGs, CE 20:3, and TG 16:0_38:6. Catalase negatively correlated with carnitine esterification, hex-cer d18:1/18:1, and hex-cer d18:2/18:0 but positively correlated with PC O-30:0 and β-alanine synthesis.

## 4 Discussion

Addressing metabolic changes in midlife women aged 30–50 after moderate training is particularly important because this life stage combines emerging insulin resistance, shift toward more central/visceral fat, decline in ovarian reserve and estrogen exposure, and often lower habitual physical activity. These interacting hormonal and body-composition changes shape long-term cardiometabolic risk and may modify how women respond metabolically to exercise in midlife (Pileggi et al., 2022). Our emerging data showed that 8 weeks of moderate physical training alter different metabolomics pathways.

Post-exercise elevation in C14:2 and C3-DC/C4-OH acylcarnitines after 8 weeks MAT may provide evidence of enhanced, yet partially incomplete, fatty-acid β-oxidation and increased mitochondrial workload during moderate aerobic exercise. In male US Army Rangers during 61-day Ranger training, plasma acylcarnitine concentrations across 10 short, medium, and long-chain species increased significantly from pre- to post-training but returned to baseline after a 2week recovery period (Baker et al., 2025). The MultiPill Exercise study showed that amino acids (AAs) and acylcarnitines (ACs) increased over 12- and 24-week physical activity interventions in multimorbid patients; baseline ACs predicted metabolic and cardiovascular improvements, while enhanced medium-chain AC mobilization after 24 weeks correlated negatively with peak power output gains, positioning these ACs as promising predictive biomarkers (Bankamp et al., 2025). The elevated C14:2 and C3-DC/C4-OH likely reflects high rates of fat and amino-acid catabolism with incomplete β-oxidation, providing the liver and muscles with additional oxidative substrates for recovery. These shifts may act as a metabolic ‘buffer’ by exporting excess acyl-CoA as acylcarnitines, preventing mitochondrial congestion and aligning with the concept of improved metabolic flexibility and energy turnover during training (Hernández-Saavedra et al., 2024).

Ceramides are often linked to insulin resistance and cardiometabolic risk. In line with this, our data show that moderate training is accompanied by increases in specific glycosylceramides (e.g., HexCer d18:1/18:1, HexCer d18:1/26:1, HexCer d18:2/18:0), alongside dynamic changes in ceramide species. We interpret these exercise-induced increases not as evidence of chronically elevated, lipotoxic sphingolipids, but as components of a transient sphingolipid remodeling response that adds both buffering and signaling dimensions to the ceramide changes (Carrard et al., 2021; Broussard et al., 2024). Glycosylceramides including trihexosylceramides (, Hex3Cer), which can decrease after exercise are generated by stepwise glycosylation of ceramide, human lipidomic studies link selected species to cardiometabolic risk as well as to favourable shifts with lifestyle interventions. However, the clinical significance of these lipidomic changes remains incompletely established, particularly in the absence of direct clinical or functional endpoints. In obesity-related insulin resistance, higher muscle and circulating C18:0-containing sphingolipids, including complex glycosphingolipids, associate with impaired insulin action, whereas combined diet and exercise programs can lower specific ceramides and hexosylceramides alongside improved glucose tolerance and reduced inflammation. In the present study, however, conventional cardiometabolic markers such as fasting glucose, insulin, HOMA-IR, triglycerides, catalase, and body weight did not significantly improve, and HDL levels decreased, underscoring the need for cautious interpretation of potential benefit. By contrast, acute and short-term exercise bouts can transiently raise circulating ceramides and glucosylceramides, suggesting that periods of high lipid flux direct part of the fatty-acid load through both ceramide and glycosylceramide pools before normalization with sustained training (Tonks et al., 2016; Carrard et al., 2021; Düsing et al., 2023; Broussard et al., 2024). In this context, higher selected HexCer d18:1/18:1, HexCer d18:1/26:1, and HexCer d18:2/18:0 after moderate training may be interpreted as evidence of transient expansion and turnover of glycosylceramide pools during increased lipid and sphingolipid flux, occurring in parallel with the ceramide response rather than necessarily indicating fixed, chronically lipotoxic glycosphingolipid accumulation. Importantly, these metabolomics shifts should not be directly equated with cardiometabolic protection, as no measures of vascular function, arterial stiffness, cardiorespiratory fitness, cardiovascular outcomes, or quality-of-life indices were assessed in this cohort of women aged 30–50 years. Considered together, these findings suggest dynamic remodeling of sphingolipid metabolism in response to training rather than definitive evidence of improved clinical status. Long-term basal levels, species-specific patterns, and their relationship to clinically meaningful endpoints remain the primary indicators of cardiometabolic risk and require further investigation in studies incorporating functional and patient-centered outcomes (Mardare et al., 2016; Reidy et al., 2020; Carrard et al., 2021; Rao et al., 2022; Düsing et al., 2023; Broussard et al., 2024).

On the other hand, training-induced increases in DG 16:0_20:4, DG O-18:2_18:2, DG 18:1_18:1 and TG 22:1_32:5, TG 16:0_38:6, TG 16:0_28:2 likely reflect enhanced glycerolipid turnover under high lipid flux, consistent with exercise-induced increases in lipolytic responsiveness and TAG cycling reported in endurance training models (Kawanishi et al., 2018; Ortlund et al., 2024). These DAG and TAG species can therefore be interpreted as lipidomic signatures of more dynamic glycerolipid metabolism compatible with greater adipose tissue lipolysis, higher VLDL-TG turnover, and redistribution of fatty acids into glycerolipids to support subsequent oxidation rather than as evidence of harmful neutral-lipid accumulation. This pattern is in line with reports that exercise can transiently raise hepatic and intra tissue TAG/DAG during recovery as a temporary storage pool for fatty acids, which buffers acute lipid overload and facilitates later oxidation and improved systemic metabolic health (Morris et al., 2015; Henderson et al., 2020).

Moderate physical training in women aged 30–50 was associated with a decrease in saturated cholesterol esters CE 14:0, consistent with the known beneficial impact of aerobic exercise on lipoprotein composition and a shift away from very short chain SFA enrichment in the cholesteryl ester fraction. In contrast, CE 20:3 increased, indicating altered incorporation of n-6 long-chain fatty acids into cholesterol esters during training-induced lipid flux. Although higher CE 20:3 has been linked to less favourable cardiovascular profiles in observational cohorts in the context of lower delta-5-desaturase activity. This isolated change is best interpreted as a marker of dynamic remodelling of cholesteryl-ester fatty-acid composition rather than as clearly beneficial or harmful in this relatively healthy, exercising cohort, especially in the absence of complementary data on 20:4-containing CE and desaturase ratios (Madan and Sawhney, 2024; Smart et al., 2025).

Physical training in middle-aged women was associated with decreases in selected phosphatidylcholines and lysophosphatidylcholine (PC O-30:2, PC O-28:0, PC O-30:0, PC 42:2, LPC 26:0). Notably, this pattern suggests increased utilization and turnover in circulating lipoproteins and possibly cellular membranes during and after exercise, consistent with training-induced phospholipid remodelling in skeletal muscle and circulation reported in endurance studies, where specific PC/LPC species change alongside improvements in mitochondrial function, oxidative metabolism, and inflammatory profiles (Mitchell et al., 2010; Newsom et al., 2016; Lee et al., 2018; Mendham et al., 2021). These changes can be viewed as transient membrane and lipoprotein remodelling that fits with concurrent TAG and CE alterations and may influence lipoprotein function and inflammatory signaling, in line with broader lysophosphatidylcholine literature describing context dependent pro-inflammatory actions that are modulated by chronic exercise (Knuplez and Marsche, 2020; Mendham et al., 2021).

The decrease in serum cystine after exercise indicates a redox-sensitive response consistent with altered cysteine/cystine handling and/or enhanced antioxidant capacity. Acute aerobic exercise has been shown to reduce circulating cysteine/cystine ratios, reflecting a transient shift in systemic redox balance that is influenced by training status. This change may represent more efficient utilization of cyst(e)ine for glutathione synthesis, increased activity of reductive pathways, or reduced oxidative stress during the post-exercise recovery period. Over repeated training bouts, such adaptations are consistent with improved redox homeostasis and greater resilience to oxidative challenges, as regular moderate-intensity training shifts the redox environment toward a more reducing state (He et al., 2016; Seifi-Skishahr et al., 2016).

The observed decrease in serine after training suggests shifts in one carbon and sulfur amino acid metabolism due to exercise stress. As serine donates one carbon units and participates in the transsulfuration pathway to produce cysteine and glutathione, its reduction may reflect increased utilization for nucleotide and methyl-group synthesis, enhanced antioxidant defence, and greater oxidative or gluconeogenic activity. This aligns with evidence that strenuous exercise lowers serine levels while raising other sulfur metabolites like cystathionine (Martínez-Reyes and Chandel, 2014; Ducker and Rabinowitz, 2017; Olsen et al., 2020; Reina-Campos et al., 2020). Overall, the simultaneous increase in cystine and decrease in serine indicate coordinated adaptations in redox balance and one carbon/sulfur amino acid metabolism during moderate training. These changes suggest that amino acid metabolism may contributes to the broader physiological adjustments enhancing oxidative capacity and stress resilience (Seifi-Skishahr et al., 2016; Zieliński et al., 2019; Olsen et al., 2020).

The rise in xanthine likely reflects increased purine breakdown due to greater ATP turnover during exercise. As muscles use ATP, resulting AMP is degraded to hypoxanthine and xanthine, which enter the bloodstream and are cleared during recovery. Thus, higher xanthine levels after training may indicate intensified energy demand and purine catabolism, followed by restoration of nucleotide balance, post exercise (Zarębska et al., 2019; Zieliński et al., 2019; Włodarczyk et al., 2020). Studies comparing trained and untrained individuals show that purine metabolites hypoxanthine, xanthine, and uric acid increase sharply with intense exercise, serving as indicators of exercise intensity and metabolic strain. With regular training, these spikes become smaller, reflecting more efficient purine metabolism, enhanced ATP resynthesis, and reliance on salvage pathways. Therefore, the rise in xanthine in this cohort likely represents a normal, transient response to increased energy demand and recovery, not a sign of disrupted nucleotide metabolism (Dudzinska et al., 2018; Zieliński et al., 2019; Włodarczyk et al., 2020).

Overall, the combined alterations in lipids, amino acids, and nucleobase metabolites indicate a coordinated adaptive response to moderate exercise in midlife women. Increases in acylcarnitines and cholesterol esters, along with shifts in cystine, serine, and xanthine, reflect transient metabolic stress involving ATP turnover, fatty-acid oxidation, redox regulation, and nucleotide metabolism processes that collectively enhance metabolic flexibility and recovery capacity (Davison et al., 2018; Grapov et al., 2019; Jaguri et al., 2023; Wong et al., 2024). Exercise metabolomics studies show that moderate or endurance training induces widespread changes across lipid, amino acid, and purine pathways, reflecting improved metabolic flexibility and fitness rather than shifts in single metabolites. In women aged 30–50, the observed metabolic pattern indicates an adaptive response to training stress, enhancing substrate switching and oxidative balance (Grapov et al., 2019; Kelly et al., 2020; Jaguri et al., 2023; Wong et al., 2024).

On the other hand, post-exercise, midlife women showed coordinated correlations between clinical traits (MET, insulin indices, BMI, HDL, catalase) and specific lipid, purine, and carnitine metabolites. Higher MET correlated positively with ceramide species Cer d18:1/16:0 and Cer d18:2/18:0 whereas no significant correlations were evident at baseline. This may suggest that greater exercise exposure is associated with higher short-chain ceramides; possibly reflecting enhanced fatty-acid mobilization and sphingolipid turnover during aerobic efforts, consistent with reports that acute bouts can transiently elevate ceramides whereas longer-term training tends to normalize ceramide profiles and support insulin sensitivity. Insulin, fasting blood glucose, and HOMA-IR correlated positively with hypoxanthine, PC O-28:0, LPC 26:0, DG 18:1_18:1, TG 16:0_38:6, CE 14:0, and TG 16:0_28:2, and negatively with carnitine esterification after training, whereas these associations were non-significant at baseline. This combination of increased purine degradation products and selected lipid species, together with reduced carnitine esterification, is compatible with impaired fuel handling and lipotoxic, purine-stressed states. BMI was positively associated with hexosyl-ceramide d18:1/26:1, the sum of MUFA-DGs, PC 32:3, and TG 16:0_38:6, aligning with evidence that adiposity is accompanied by glycosphingolipid accumulation and altered glycerolipid composition (Chaurasia and Summers, 2021; Bernal et al., 2025). Pre- and post-exercise, triglycerides correlated positively with a cluster of MUFA-DGs and CE 14:0, suggesting that mobilized fatty acids are more likely to be directed into storage lipids rather than fully oxidized. The repeated co-variation of TG 16:0_38:6 and DG 18:1_18:1 with total TG points to a MUFA-dominant lipid signature that may index post-exercise lipid handling in these women. Notably, an inverse relationship with serine emerged only after training, with no correlation at baseline, further hinting that greater glycerolipid accumulation is accompanied by altered amino acid-lipid partitioning, potentially drawing serine away from other metabolic pathways (Handzlik et al., 2023; Jaguri et al., 2023). HDL showing positive correlation with hexosyl-ceramide d18:1/16:0 that was not apparent at baseline. This may reflect a shift toward a less adverse ceramide profile in line with reports linking specific ceramide species to cardiometabolic risk. However, given the absence of improvements in conventional cardiometabolic markers and the lack of clinical or quality-of-life outcomes in this cohort, the direction and clinical relevance of this association remain uncertain. In parallel, the persistent negative correlations between HDL and xanthine, MUFA-DGs, and TG 16:0_38:6 both before and after training indicate that individuals with higher HDL tended to have lower purine-related stress and fewer potentially unfavourable MUFA-rich triglyceride/diacylglycerol species across the study period.

Together, these patterns support the idea that exercise may remodel the HDL centered metabolic network, even in the context of a modest reduction in HDL concentration, potentially yielding a qualitatively “healthier” HDL-associated metabolic profile (Hilvo et al., 2020). In parallel, catalase activity associated inversely with carnitine esterification and selected hexosyl-ceramides with no significant correlation at baseline. Additionally, catalase positively correlated with PC O-30:0 and β-alanine-linked features poste exercise only, suggesting that post-exercise antioxidant responses may coincide with shifts in sphingolipid and β-alanine-related metabolism (Yang and Wu, 2025).

## 5 Conclusion

This exploratory study describes a coordinated serum metabolomic response to 8 weeks of moderate aerobic training in women aged 30–50 years, spanning multiple metabolite classes including ceramides, glycosylceramides, acylcarnitines, glycerolipids, phospholipids, amino acids, and xanthine. The findings suggest female-specific remodeling of ceramide, glycosphingolipid, and MUFA-glycerolipid networks, with purine–lipid species linked to insulin resistance, BMI, MET, and HDL. However, these results are hypothesis-generating, based on circulating metabolites alone, and their clinical relevance for cardiometabolic health in midlife women requires validation in larger, longer-term studies with direct functional and clinical outcomes.

## 6 Limitation

This study has several limitations. First, the small sample size and the fact that the cohort consisted exclusively of midlife women from a single geographic region limit statistical power and generalizability. Future studies should include larger and more diverse populations (e.g., broader BMI ranges, different ethnic backgrounds, and men) to improve translational relevance. Second, the absence of a parallel non-exercise control group limits causal inference; although we assessed within-participant changes before and after the intervention, temporal, dietary, and environmental factors may also have contributed to the observed metabolic changes. Third, we recognize that hormonal status, diet, sleep, and other lifestyle factors may influence lipid- and amino acid-related metabolites. Although all participants were premenopausal, menstrual cycle phase and hormonal profiles were not systematically characterized or controlled, which may affect mechanistic interpretations and limit the generalizability of our findings. Fourth, physical activity outside the supervised exercise sessions was assessed using IPAQ self-report data, which are subject to recall bias and measurement error. Fifth, the intervention duration was limited to 8 weeks, which may capture early metabolic adaptations but is insufficient to characterize long-term remodeling of lipid and sphingolipid pathways. Sixth, the study did not include direct assessments of quality of life, physical function, or cardiometabolic outcomes (e.g., blood pressure, vascular imaging, endothelial function, arterial stiffness, echocardiography, and cardiorespiratory fitness). Notably, several conventional cardiometabolic parameters, including fasting glucose, insulin, HOMA-IR, triglycerides, catalase, and body weight, did not significantly improve, and HDL-cholesterol decreased. Consequently, the clinical significance of the observed metabolomic shifts remains uncertain and requires further investigation in future studies. Finally, the metabolomic analyses were exploratory and involved multiple comparisons. Although appropriate statistical corrections were applied, these findings should be considered hypothesis-generating and require validation in independent cohorts.

## Data Availability

The raw data supporting the conclusions of this article will be made available by the authors, without undue reservation.
